# Ribosomal DNA transcription in prefrontal pyramidal neurons is decreased in suicide

**DOI:** 10.1007/s00406-019-00996-0

**Published:** 2019-03-11

**Authors:** Marta Krzyżanowska, Johann Steiner, Dorota Pieśniak, Karol Karnecki, Michał Kaliszan, Marek Wiergowski, Krzysztof Rębała, Ralf Brisch, Katharina Braun, Zbigniew Jankowski, Monika Kosmowska, Joanna Chociej, Tomasz Gos

**Affiliations:** 1 Department of Forensic Medicine, Medical University of Gdańsk, ul. Dębowa 23, 80-204 Gdańsk, Poland; 2 Department of Psychiatry, Otto-von-Guericke-University, Magdeburg, Germany; 3 Department of Zoology/Developmental Neurobiology, Institute of Biology, Otto-von-Guericke-University, Magdeburg, Germany

**Keywords:** Postmortem, Suicide, Prefrontal cortex, AgNOR staining

## Abstract

Prefrontal cortical regions, which are crucial for the regulation of emotionally influenced behaviour, play most probably a dominant role in the pathogenesis of suicide. The study was carried out on paraffin-embedded brain tissue blocks containing specimens from the anterior cingulate cortex (dorsal and ventral parts), the orbitofrontal cortex, and the dorsolateral cortex obtained from 23 suicide completers (predominantly violent) with unknown psychiatric diagnosis and 25 non-suicidal controls. The transcriptional activity of ribosomal DNA (rDNA) as a surrogate marker of protein biosynthesis was evaluated separately in layers III and V pyramidal neurons in regions of interest (ROIs) mentioned above by the AgNOR silver staining method bilaterally. The overall statistical analysis revealed a decrease of AgNOR area suggestive of attenuated rDNA activity in suicide victims versus controls, particularly in male subjects. Further ROI-specific *post-hoc* analyses revealed decreases of the median AgNOR area in suicides compared to non-suicides in all 16 ROIs. However, this effect was only significant in the layer V pyramidal neurons of the right ventral anterior cingulate cortex. Our findings suggest that decreased rDNA transcription in prefrontal pyramidal neurons plays possibly an important role in suicide pathogenesis.

**Electronic supplementary material:**

The online version of this article (10.1007/s00406-019-00996-0) contains supplementary material, which is available to authorized users.

## Introduction

Disturbances of the prefrontal cortex (PFC) regions, which play a pivotal role in behavioural regulation, are implicated in a multifaceted way in suicidal behaviour (for reviews see: [1, 2]). This behaviour has been proposed to be an independent mental disorder in the fifth edition of the Diagnostic and Statistical Manual of Mental Disorders—DSM V [3] in accordance with numerous neurobiological research data (for reviews see: [1, 2]). The distinctness of suicide in mental disorders has been also suggested by our own neuropathological research [4–6] (for a review of our previous studies see: [7]).

Nucleolar organising regions (NORs) are genetic loci on chromosomes that are composed of ribosomal DNA (rDNA) and proteins, some of which are argyrophilic. In human interphase cells, silver-stained NORs (AgNORs) clustered together in the nucleolus represent the site of transcriptionally active NORs and ribosomal RNA synthesis, which constitutes approximately half of the entire transcriptional activity in the cell. In the AgNOR staining evaluated by light microscopy, AgNORs are indistinguishable from each other and form the AgNOR area. It is located in the nucleolar area but smaller than this area (compared, for instance, with haematoxylin-eosin and Nissl stainings [8]). As a surrogate marker of protein biosynthesis and an important sensor of cellular stress of different nature, the transcriptional activity of rDNA can be assessed by measuring AgNOR parameters. These are: AgNOR area (representing the nucleolus), AgNOR number (i.e. the number of AgNOR areas within one nucleus) and AgNOR ratio defined as the quotient of total AgNOR area in the nucleus and nuclear area [9–22] (for reviews see: [23, 24]).

A key role of rDNA transcriptional activity in neuronal plasticity has been proven in neuronal culture [25–27] and molecular studies have revealed that this activity is decreased in the hippocampus of suicidal patients [28]. Our previous AgNOR studies of the PFC [13, 15] and other brain structures [5–7, 10, 14, 17] have suggested decrease of neuronal rDNA transcription in suicide, which is consistent with molecular results [28]. In addition, our recent AgNOR research on dorsal raphe nucleus (DRN) neurons has implied a decreased rDNA transcription as a diagnosis-overreaching phenomenon specific for suicide [5, 6], which may be useful for the forensic differentiation between suicidal and non-suicidal death [6]. Previous neuropathological research by us and other researchers has also suggested an increased microglial reaction in the PFC of suicidal patients as a phenomenon independent of psychiatric diagnosis [29, 30]. This finding may be related to decreased rDNA transcription in pyramidal neurons via the induction of oxidative stress [31, 32].

Therefore, in the present study we hypothesized a decreased rDNA transcriptional activity in prefrontal pyramidal neurons in suicide completers regardless of their underlying psychiatric diagnosis (i.e. independent of psychiatric comorbidity) and tested this hypothesis by the application of AgNOR staining in forensic postmortem material. We aimed at both basic research on the neurobiology of suicide and the evaluation of a possible usefulness of this method in forensic diagnostics for the differentiation between suicidal and non-suicidal death.

## Materials and methods

### Human brain tissue

Prefrontal parts of both hemispheres of sudden death controls and suicide victims with unknown data both on psychiatric comorbidity and on possible psychotropic medication preceding death (typical for most of suicide cases autopsied in our Department of Forensic Medicine) were obtained during routine forensic autopsies in accordance with existing EU law regulations. The study has been approved by the local ethics committee of the Medical University of Gdańsk in accordance with the ethical standards laid down in the Declaration of Helsinki, version 1989.

Detailed diagnostic, demographic and toxicological data are presented in Supplementary Table 1. Violent suicide methods prevailed in the suicide cohort (15 out of 23), which is representative for our autopsy material. All brains were free of neuropathology suggestive of vascular, traumatic, inflammatory, neoplastic and neurodegenerative processes. A toxicology screen on blood and urine for ethanol was performed at each autopsy. The majority of investigated cases (19 suicide victims and 18 controls, see Supplementary Table 1) revealed a blood alcohol concentration (BAC) below the limit of quantification (LOQ), i.e. <0.2 g/l according to internationally accepted analytical guidelines. Other substances of abuse, antidepressant and antipsychotic drugs, as well as their metabolites were investigated when an intoxication was suggested by the scene inspection and/or other available information sources prior to the autopsy, i.e. in eight cases. These cases constituted the non-violent suicide subgroup (see Supplementary Table 1).

**Fig. 1:**
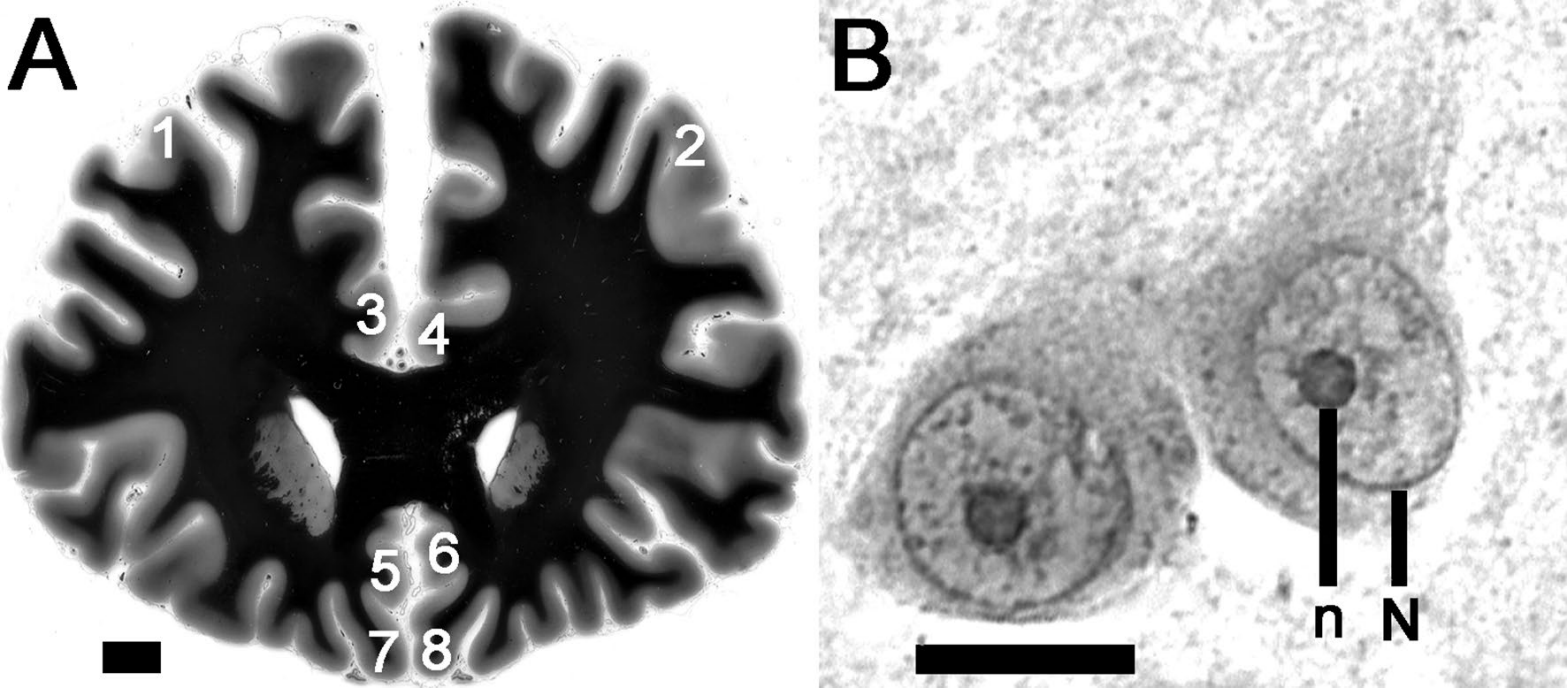
**a** Regions of interest in the prefrontal cortex are outlined bilaterally (1—dorsolateral right, 2—dorsolateral left; 3—anterior cingulate dorsal right, 4—anterior cingulate dorsal left; 5—anterior cingulate ventral right, 6—anterior cingulate ventral left; 7—orbitofrontal right, 8—orbitofrontal left) at low magnification picture of the Nissl-myelin-stained coronal section at the level where investigations were carried out (scale bar 10 mm; the staining method is described in [33]).** b** After AgNOR staining the borders of the nucleus (N) and the AgNOR area (representing the nucleolus) (n) are clearly visible within prefrontal pyramidal neurons (scale bar 10 μm)

Prefrontal parts of the brains were separated from both hemispheres by coronal sections at the level of temporal poles and fixed *in toto* in 10% phosphate-buffered formaldehyde for 1 week. After being fixated, tissue blocks were isolated closely to the section plane from the following prefrontal regions: dorsolateral prefrontal cortex (DLPFC), anterior cingulate cortex (dorsal (ACd) and ventral (ACv) parts) and orbitofrontal cortex (OFC) (see Fig. 1a), and embedded in paraffin. Subsequently, serial 5-µm thick transverse sections in frontal plane were cut along these tissue blocks. Every 200th section was mounted and stained for AgNOR.

### AgNOR staining

Silver staining was carried out as previously described [10]. Briefly, paraffin sections were dewaxed and rehydrated through graded alcohols. The silver stain was freshly prepared by mixing 2% gelatin (dissolved in 1% aqueous formic acid) with 50% aqueous silver nitrate solution at a 1:2 ratio. The sections were incubated with this mixture in a dark moist chamber at room temperature for 45 min and subsequently washed with deionised water. Following this protocol, the AgNOR area—containing AgNORs (that are clustered, undistinguishable from each other) and representing the nucleolus—appears as an intranuclear, clearly delineated black or dark brown small spot and the nuclear border is clearly visible in the majority of large pyramidal neurons (Fig. 1b). Glia cells were distinguished from neurons according to established cytomorphological criteria [34].

### Quantification

In each of 16 investigated regions of interest (ROIs), i.e. cortical layers III and V in each of 4 prefrontal regions bilaterally, AgNOR parameters were determined in 40 neurons with clearly visible borders of the nucleus and AgNOR areas. These neurons were selected throughout available AgNOR stained sections, i.e. in five sections for each tissue block (40 sections for each case), on the average. Thus, the AgNOR parameters of 640 pyramidal neurons were investigated in each case. The number of investigated neurons was established arbitrarily in accordance with diagnostic and research studies employing the AgNOR method. This method does not require an estimation of the number of cells and/or nuclei [10]. The neurons were sampled using a 400×magnification. The AgNOR areas (composed of clustered AgNORs and representing the nucleoli), their number and the nuclear area within a single sampled neuron were determined using a light microscope attached to a computer image analysis system (cellSens®, Olympus, Japan). In this system, each of the neurons sampled by 400×magnification was visualised digitally, focused, and the sharpest and longest profiles of the nucleus and AgNOR areas were traced by the mouse cursor on the screen. As a result, the numerical values of AgNOR and nuclear areas and the numbers of AgNOR areas were calculated automatically. Subsequently, the AgNOR ratio (relative AgNOR area) was derived by dividing the total AgNOR area by the nuclear area, taking into account all the AgNOR areas present per neuronal nucleus. This procedure was performed separately for each of the sampled neurons. The sampled measures were averaged to derive a single set of values for each ROI in each of investigated cases (i.e. 16 values in each case).

### Data analysis

Statistical analyses were performed with the data analysis software system STATISTICA version 10 (StatSoft®, Inc. 2011, http://www.statsoft.com). As normal distribution was not given for all analysed AgNOR parameters (i.e. significant values of Kolmogorov–Smirnov tests were obtained), non-parametric statistical procedures were used in hierarchic mode.

First, STATISTICA generalized linear/nonlinear models (GLZ) module containing general custom designs (GCD) procedure was applied as an omnibus method to analyse associations between dependent variables (i.e. AgNOR parameters) and independent categorical variables (i.e. suicidal/control groups, PFC region, cortical layer, and sex as the categorical confounding variable). The results of GCD analysis were reported automatically including the Wald statistic value, degrees of freedom and the respective *P* value. This initial GCD analysis revealed that only AgNOR area was related to suicide, which was an effect associated with sex. Therefore, further analyses were performed only for AgNOR area.

Age, postmortem interval, brain weight and BAC (values below LOQ were accounted null values in statistical analysis) were considered as numerical confounding variables. Therefore, the subsequent GCD procedure was applied in each of ROIs to analyse associations between AgNOR area and these variables. Supplementary to GCD analyses in ROIs, Spearman correlation coefficients were calculated to determine the impact of numerical variables which might confound AgNOR area.

Subsequently to GCD analyses, unadjusted two-way *post-hoc* comparisons with Mann–Whitney *U* test and the *χ*^*2*^ test were used to detect the possible differences between the study groups with respect to the variables mentioned above (i.e. AgNOR area and confounders). *U* test was also used to detect the possible difference in AgNOR area between violent (*n* = 15) and non-violent suicides (i.e. self-poisoning cases in our cohort, *n* = 8). All statistical tests were two-tailed. Generally, *P* values of 0.05 were accepted as statistically significant.

Kruskal–Wallis analysis of the variance of ranks (*H* test) with subsequent *post-hoc U* tests were performed for the evaluation of differences in the AgNOR area related to sex between suicides and controls, which was suggested by the GCD analysis; in this procedure *U* test *P* values were adjusted to multiple comparisons according to the Bonferroni correction.

STATISTICA automatic neuronal networks (SANN) module containing receiver operating characteristic (ROC) procedure was applied for the evaluation of the discriminative value of the AgNOR method, i.e. its accuracy (represented by the area under ROC curve), sensitivity and specificity for the differentiation between suicidal and non-suicidal death.

## Results

### Qualitative analysis of the AgNOR staining

After AgNOR staining of the PFC pyramidal neurons, borders of the AgNOR area (containing AgNORs that were clustered together and indistinguishable from each other) were clearly visible (Fig. 1b) in line with the staining patterns in these neurons presented previously [13, 15]. Most of the neurons contained one AgNOR area (representing the nucleolus in this staining method). Two or more AgNOR areas were observed very rarely, which explains why the AgNOR number was near 1.

### Quantitative analysis of the AgNOR staining

The differences in AgNOR parameters were beyond qualitative evaluation and they could only be captured by means of quantitative measurements.

Cumulative analyses of results from all investigated 16 ROIs (i.e. from cortical layers III and V in 4 evaluated PFC regions bilaterally, 384 suicidal and 400 control values for each of AgNOR parameters) by GCD procedure revealed the strong association of forensic diagnosis (i.e. suicide versus non-suicide) with the AgNOR area (Wald statistic = 33.87, *df* = 1, *P* = 0.00000000589; median values 6.91 µm^2^ and 7.21 µm^2^ for suicides and non-suicidal controls, respectively, see Table 1). This effect was not associated with the brain hemisphere, investigated PFC region or cortical layer. Instead, it was closely associated with sex (Wald statistic = 37.21, *df* = 1, *P* = 0.0000000107), i.e. mainly driven by male subjects (see next paragraph). Other AgNOR parameters were not associated with suicide/non-suicide dichotomy (insignificant Wald statistic values) and, therefore, excluded from further analyses. As revealed by the ROC curve for cumulated AgNOR area values, the method accuracy (represented by the area under ROC curve, AUC) was 58%; its sensitivity according to suicide detection was 27%, whereas the specificity of suicide exclusion (i.e. the detection of non-suicidal case) was 82%.

**Table 1:** Summarised statistical data regarding the comparison between suicide victims (*n* = 23) and controls (*n* = 25)

Intergroup comparisons	Sex	Age (year)	PMI (h)	BAC (g/l)	AgNOR area cum all	AgNOR area cum males	AgNOR area cum females	AgNOR area right ACv layer V
Suicide victims								
Ratio/median *(q1, q3)*	17 m/6f	41.5 (28.5, 57.5)	24 (24, 39)	0.00 (0.00, 0.00)	6.914 (6.228, 7.781)	6.698 (6.040, 7.448)	7.558 (6.895, 8.997)	6.845 (6.148, 7.515)
Controls								
Ratio/median *(q1, q3)*	21 m/4f	57 (28, 61)	24 (15, 30)	0.00 (0.00, 0.00)	7.212 (6.452, 8.418)	7.170 (6.406, 8.333)	7.744 (6.849, 8.992)	7.734 (7.064, 8.368)
Statistics								
Test	*χ*^*2*^-test	*U*	*U*	*U*	*GCD*	*GCD*	*GCD*	*U*
Characteristic value	*χ*^*2*^ = 0.610	*Z* = 0.890	*Z* = − 0.790	*Z* = 0.420	*Wald statistic* = 33.87	*Wald statistic* = 43.31	*Wald statistic* = 0.03	*Z* = 2.270
*P* value	0.435	0.373	0.429	0.675	**0.00000000589**	**0.0000000000468**	0.866	**0.0232**

*PMI*—postmortem interval, *BAC*—blood alcohol concentration, *cum*—cumulated results from layers III and V in all analysed regions of interest (ROIs), *ACv*—anterior cingulate cortex, ventral part, *f*—female, *m*—male, *q1* and *q3*—quartile 1 and 3; *GCD*—general customs design procedure (in generalized linear/nonlinear models of data analysis software system STATISTICA). (See Supplementary Table 1 for the detailed values of confounding variables and AgNOR parameters in the right ACv layer V, i.e. the only ROI where the significant difference in AgNOR area in pyramidal neurons was found between compared groups.)

In accordance with initial GCD analysis, further ROI-specific analyses by *post-hoc U* tests revealed decreases of the median AgNOR area in suicides compared to non-suicides in all 16 ROIs. However, this effect was only significant in the right ACv pyramidal cells layer V (see Table 1). No significant difference of AgNOR area existed between violent and non-violent suicide victims in both the cumulative analysis of the data and in the ROI-specific analyses (non-significant *U* test *P* values).

### Confounders

The suicidal versus control group were matched regarding sex (non-significant *χ*^*2*^ test *P* value, see Table 1). No significant intra-group differences between sexes were found in the AgNOR area in the right ACv pyramidal cells layer V (non-significant *U* test *P* values) despite higher median values presented by females. However, according to the associated effect of forensic diagnosis and sex suggested by the initial GCD procedure, only males revealed significant difference between suicides and controls in this ROI (*U* test *P* = 0.0256; for a comparison see Table 1). On the other hand, significant intra-group differences between sexes were found in the cumulative analysis of AgNOR area values from all ROIs (corrected *U* test *P* values 0.0048 in control and 0.00000000013 in suicidal group; females presented higher values). GCD cumulative analyses calculated separately for both sexes revealed more significant difference in this parameter between suicides and controls in males compared to the analysis of all cases, whereas no significant effect was observed in females (see Table 1). Therefore, the observed phenomenon was specific for males. Nevertheless, ROC values for males (AUC 62%, sensivity 28%, and specifity 82%) were similar to those obtained for the entire cohort.

Age, PMI and BAC revealed no significant differences between suicides and controls (non-significant *U* test *P* values, see Table 1), similarly as the brain weight. ROI-specific analyses by the GCD procedure revealed no associations between these numerical confounders and AgNOR area (non-significant Wald statistic *P* values) in suicides and controls in any of investigated ROIs. Correspondingly, no relevant correlations were found between numerical confounders and AgNOR area in any ROI in both groups (non-significant Spearman correlation *P* values and/or irrelevant *r* values). Therefore, the results of AgNOR area analysis were not confounded by these variables.

## Discussion

Our study revealed the decreased AgNOR area suggestive of attenuated rDNA transcription in prefrontal pyramidal neurons in suicide victims versus controls. Interestingly, the observed abnormality was specific for male suicides. However, the interpretation of this finding is not unequivocal. On the one hand, this effect could be related to sex-specific differences in chronically disturbed neuronal plasticity, according to reports on animal models (for a review see: [35]) and emerging human molecular studies [36] of mental disorders. On the other hand, it could be related to the higher number of males in both suicidal (17 out of 23) and control (21 out of 25) cohorts of our study. This issue could be resolved by further research on cohorts with more numerous female subjects.

The observed AgNOR area decrease was not influenced by other potentially confounding variables, among them postmortem interval. The significance was accentuated in the cumulative evaluation of all analysed ROIs independently on the hemisphere, region, and layer (i.e. layer III or V). This phenomenon could be related to characteristics of PFC regions, which cooperate reciprocally in functional network [37]. However, despite the fact that the AgNOR area was decreased in suicides in all ROIs, the right ACv layer V was the only ROI where the significant difference between suicides and controls was found in separate *post-hoc* analyses. This effect could be related to the outstanding role played by the ventral AC in the regulation of emotionally influenced behaviour [38], which is profoundly disturbed in processes associated with suicide [13, 39].

The results are in line with our previously published findings, which suggested a decreased rDNA transcription in PFC pyramidal neurons specific for depressed suicide completers compared to non-suicidal patients from both major depressive disorder and bipolar disorder diagnostic groups of affective disorders [13, 15]. Therefore, both our previous and present results may suggest decreased rDNA transcription in prefrontal pyramidal neurons in suicide regardless of psychiatric comorbidity. Despite of the statistical significance of our results, the AgNOR area values overlapped between the suicide and non-suicide cohorts. Consequently, the method accuracy (AUC = 56%) does not suggest usefulness for the forensic differentiation between suicidal and non-suicidal death [40] in opposite to our previous study of the DRN (AUC = 89%) [6].

A multitude of factors influence rDNA transcription, taking into account that the expression of hundreds of genes is involved in the regulation of this fundamental molecular process [41]. The bottleneck of these factors is the mammalian target to rapamycin (mTOR) intracellular signaling pathway, which plays the most important regulatory role in rDNA transcription [42]. Many molecular signatures of suicide are involved directly or indirectly in the action on this pathway. An outstanding example is the abnormality of glucocorticoid receptor (GR), a key component of the disturbed stress axis, which is decreased in the PFC in suicide (for a review see [43]). The decreased gene expression of spindle and kinetochore associated complex subunit 2 (SKA2) involved in the transport of GR from the neuronal cytoplasm to the nucleus may significantly contribute to this dysfunction [44]. Moreover, GR is negatively influenced by polyamines [45], which are multipotent regulatory molecules increased in suicidal PFC [46]. The dysfunction of GR produced by cumulative factors may in turn lead to the disturbed mTOR function in neurons [47] with a subsequent decrease in rDNA transcription.

Disturbed glutamatergic transmission in the PFC of suicide victims as suggested by previous postmortem studies [48–50] may also lead to mTOR hypoactivity in pyramidal neurons [51]. The blockade of *N*-methyl-d-aspartate (NMDA) glutamate receptors on prefrontal GABAergic interneurons with subsequent disinhibition of glutamatergic transmission, brain-derived neurotrophic factor (BDNF) gene expression and mTOR activity in pyramidal neurons seems to be the most important molecular effect in the rapid suicide-preventive action of ketamine [51].

Our previous research suggested GABAergic hyperactivity in prefrontal regions in depressed suicides [52], which may lead to the decreased rDNA transcription in prefrontal pyramidal neurons via the suppressed expression of BDNF gene [53, 54]. BDNF constitutes a potent activator of neuronal mTOR [51] and, therefore, also rDNA transcriptional activity. Thus our current results correspond with previous molecular studies, which revealed decreased BDNF levels (for a review see: [55]) and mTOR signaling [56] in the suicidal PFC. Moreover, the disturbed serotonergic system morphology in suicide observed by us [5, 6, 10] and other researchers [57] (for a review see: [58]) may be also related to BDNF hypofunction [59] (for reviews see: [60, 61]).

Previous neuropathological research by our workgroup [29] and others [30] suggested an increased microglial reaction in prefrontal regions as a diagnosis-overreaching phenomenon specific for suicide. The increased levels of pro-inflammatory cytokines found in prefrontal regions and cerebrospinal fluid of suicide victims suggested the devastating neurodegenerative role of microglia activation in suicide [62]. As revealed by experimental studies, activated microglia may induce oxidative stress in target neurons [31]. Besides microglia, oxidative stress in pyramidal neurons may be induced by 5HT2A serotonergic receptors (5HT2ARs) [63], which are up-regulated in the PFC of suicidal patients [64] (for reviews see: [43]). Therefore, both microglia activation and 5HT2ARs up-regulation in the PFC may contribute to the decreased rDNA transcription in pyramidal neurons in suicide in a mTOR-dependent manner by the oxidative stress-induced protein REDD1, which is a potent mTOR inhibitor [47, 65].

Alternatively, human postmortem data suggest that the decreased rDNA transcription in PFC pyramidal neurons may be also a consequence of the hypermethylation of rDNA promoter region independently on mTOR pathway [28, 66, 67]. On the other hand, the hypermethylation of BDNF promoter was revealed in the neocortex of suicide victims [67, 68]. Therefore, the epigenetic phenomena might hypothetically influence the rDNA transcription in pyramidal neurons in both direct and indirect manner.

Multiple upstream factors lead to attenuated rDNA transcription and disturbed plasticity of prefrontal pyramidal neurons is a far-reaching deleterious consequence of this devastating molecular phenomenon [25]. Therefore, our current results are in line with previous neuropathological study, which observed a decrease in pyramidal dendrites in the PFC of depressed suicide victims [69]. Since the arborisation of the pyramidal dendritic tree is a fundamental morphological signature of neuronal plasticity crucial for PFC function, the activation of diminished rDNA transcription targeted specifically in PFC pyramidal neurons may constitute new therapeutic avenue in future suicide prevention.

## Limitations

The present study has certain limitations that have to be considered: (1) A relatively small number of predominantly male cases have been analyzed. Therefore, results have to be confirmed in a larger sample with more numerous female subjects. (2) The psychiatric diagnoses (also according to substance use disorders) and the data on possible psychotropic medication preceding death were not available. The levels of psychotropic drugs were established only in eight suicide victims where medication overdose constituted a cause of death. However, our current study did not aim at the relation between suicide and other mental disorders and our previous studies did not suggest that the decreased AgNOR area in prefrontal pyramidal neurons may be related to the medication in the last 3 months of life [13, 15]. (3) The application of paraffin-embedded tissue is a limitation of our method compared to frozen brain samples, which would allow the application of a wider set of approaches.

## Conclusion

In summary, our results suggest the decreased rDNA transcription in pyramidal PFC neurons in suicide victims as a presumable consequence of multiple molecular events. The present method could not probably aid forensic differentiation diagnostics between suicidal and non-suicidal death in cases where traditional methods are inconclusive. However, further research is warranted to appropriately evaluate this issue.

## Electronic supplementary material

Below is the link to the electronic supplementary material.

Supplementary material 1 (DOCX 31 KB)
