# Supplementary material for: Ribosomal DNA transcription in prefrontal pyramidal neurons is decreased in suicide
Source: Eur Arch Psychiatry Clin Neurosci. 2019 Mar 11;270(7):859–67. doi: 10.1007/s00406-019-00996-0 (PMC7474709; doi:10.1007/s00406-019-00996-0)
Supplement: Supplementary file 1 — Supplementary material 1 (DOCX 31 KB) [file 406_2019_996_MOESM1_ESM.docx]

**Ribosomal DNA transcription in prefrontal pyramidal neurons is decreased in suicide**

Marta Krzyżanowska, Johann Steiner, Dorota Pieśniak, Karol Karnecki, Michał Kaliszan, Marek Wiergowski, Krzysztof Rębała, Ralf Brisch, Katharina Braun, Zbigniew Jankowski, Monika Kosmowska, Joanna Chociej, Tomasz Gos

**European Archives of Psychiatry and Clinical Neuroscience**

Corresponding author:

Tomasz Gos, MD, PhD

Department of Forensic Medicine

Medical University of Gdańsk

ul. Dębowa 23

80-204 Gdańsk, Poland

E-mail: [gost@gumed.edu.pl](mailto:gost@gumed.edu.pl)

**Supplementary Table 1** Detailed diagnostic and demographic data and the values of parameters revealed by AgNOR staining in the right ACv (anterior cingulate cortex, ventral part) pyramidal cells layer V, where the significant decrease of AgNOR area was found in suicide victims (n=23) compared to control subjects (n=25). *Abbreviations:* f – female; m – male; *q1* and *q3* – quartile 1 and 3; PMI – postmortem interval; BAC – blood alcohol concentration; LOQ – limit of quantification (BAC = 0.2 g/l).

| Case ID | Cause of death | Sex | Age [yr] | PMI [h] | BAC [g/l] | Nuclear area (µm^2^) | AgNOR area (µm^2^) | AgNOR number | Relative AgNOR area |
| --- | --- | --- | --- | --- | --- | --- | --- | --- | --- |
|  |  |  |  |  |  |  |  |  |  |
|  | SUICIDE VICTIMS |  |  |  |  |  |  |  |  |
| 1 | Hanging | m | 54 | 24 | <LOQ | 113.960 | 6.127 | 1.000 | 0.053 |
| 2 | Hanging | m | 22 | 24 | <LOQ | 113.827 | 6.169 | 1.000 | 0.054 |
| 3 | Hanging | m | 62 | 24 | <LOQ | 85.439 | 7.204 | 1.000 | 0.084 |
| 4 | Hanging | m | 38 | 24 | <LOQ | 88.934 | 4.664 | 1.025 | 0.053 |
| 5 | Hanging | f | 38 | 36 | <LOQ | 114.961 | 4.694 | 1.013 | 0.042 |
| 6 | Hanging | m | 29 | 24 | 0.80 | 90.445 | 6.757 | 1.000 | 0.075 |
| 7 | Hanging | f | 57 | 24 | <LOQ | 81.544 | 7.330 | 1.000 | 0.091 |
| 8 | Hanging | m | 31 | 12 | <LOQ | 92.202 | 10.733 | 1.000 | 0.115 |
| 9 | Hanging | m | 43 | 24 | <LOQ | 86.455 | 6.234 | 1.000 | 0.075 |
| 10 | Hanging | m | 13 | 48 | <LOQ | 69.585 | 6.072 | 1.000 | 0.089 |
| 11 | Hanging | m | 42 | 6 | <LOQ | 96.868 | 5.914 | 1.000 | 0.063 |
| 12 | Hanging | m | 25 | 66 | 1.91 | 100.984 | 7.858 | 1.000 | 0.079 |
| 13 | Hanging | m | 19 | 24 | <LOQ | 120.901 | 10.164 | 1.000 | 0.085 |
| 14 | Hanging | m | 47 | 24 | <LOQ | 94.895 | 7.417 | 1.000 | 0.080 |
| 15 | Self-harm by sharp object (stab wound of head) | m | 41 | 72 | <LOQ | 98.855 | 8.109 | 1.000 | 0.083 |
| 16 | Self-poisoning  (quetiapine) | m | 59 | 12 | 2.40 | 101.476 | 7.433 | 1.000 | 0.073 |
| 17 | Self-poisoning (alprazolam, mianserin, verapamil) | f | 85 | 24 | <LOQ | 102.218 | 7.596 | 1.000 | 0.076 |
| 18 | Self-poisoning (zopiclone) | m | 58 | 48 | <LOQ | 82.036 | 5.283 | 1.000 | 0.064 |
| 19 | Self-poisoning (olanzapine, clomipramine, amlodipine, bisoprolol) | f | 65 | 24 | <LOQ | 88.388 | 6.686 | 1.000 | 0.076 |
| 20 | Self-poisoning  (chlorprothixen) | m | 53 | 42 | <LOQ | 86.704 | 7.028 | 1.000 | 0.085 |
| 21 | Self-poisoning  (morphine) | m | 28 | 12 | <LOQ | 90.477 | 6.534 | 1.000 | 0.073 |
| 22 | Self-poisoning (venlafaxine, trazodone, tramadol) | f | 33 | 48 | 0.35 | 79.923 | 6.933 | 1.000 | 0.087 |
| 23 | Self-poisoning  (clozapine) | f | 68 | 24 | <LOQ | 107.228 | 6.292 | 1.000 | 0.060 |
| *Suicide victims: ratio/median (q1, q3)* | | *17m/6f* | *41.5 (28.5, 57.5)* | *24 (24, 39)* | *0.00 (0.00, 0.00)* | *93.549 (86.579, 104.723)* | *6.845(6.148, 7.515)* | *1.000 (1.000, 1.000)* | *0.076 (0.064, 0.084)* |
|  |  |  |  |  |  |  |  |  |  |
|  | CONTROLS |  |  |  |  |  |  |  |  |
| 24 | Sudden cardiac death | m | 61 | 24 | <LOQ | 111.916 | 10.801 | 1.050 | 0.101 |
| 25 | Traffic accident (motorcycle rider, injury of subclavian artery and traumatic haemothorax) | m | 25 | 12 | <LOQ | 94.199 | 9.218 | 1.000 | 0.098 |
| 26 | Right ventricular failure | m | 58 | 48 | <LOQ | 102.995 | 9.114 | 1.000 | 0.091 |
| 27 | Sudden cardiac death | m | 63 | 15 | <LOQ | 111.103 | 8.191 | 1.000 | 0.076 |
| 28 | Traffic accident (pedestrian injured in collision with motorcycle, multiple injuries) | m | 24 | 12 | <LOQ | 114.914 | 9.433 | 1.000 | 0.083 |
| 29 | Pulmonary embolism (acute corpulmonale) | f | 59 | 24 | <LOQ | 96.919 | 9.871 | 1.000 | 0.102 |
| 30 | Traffic accident (car occupant, multiple injuries) | m | 20 | 24 | <LOQ | 87.766 | 7.654 | 1.000 | 0.087 |
| 31 | Traffic accident (car occupant, multiple injuries) | f | 59 | 24 | <LOQ | 80.114 | 8.044 | 1.000 | 0.104 |
| 32 | Traffic accident (pedestrian injured in collision with motorcycle, multiple injuries) | m | 24 | 12 | 0.80 | 93.192 | 7.734 | 1.000 | 0.084 |
| 33 | Sudden cardiac death | m | 45 | 12 | <LOQ | 95.542 | 8.368 | 1.000 | 0.088 |
| 34 | Stabbed (open wounds of thorax) | m | 57 | 10 | 1.70 | 97.349 | 7.759 | 1.000 | 0.079 |
| 35 | Transport accident (pedestrian injured in collision with railway vehicle, injury of cervical spinal cord) | f | 19 | 20 | <LOQ | 102.671 | 7.763 | 1.000 | 0.072 |
| 36 | Stabbed (open wound of thorax) | m | 28 | 7 | 3.15 | 92.865 | 8.327 | 1.000 | 0.089 |
| 37 | Traffic accident (pedestrian injured in collision with car, multiple injuries) | m | 71 | 24 | 0.74 | 99.372 | 7.391 | 1.000 | 0.076 |
| 38 | Sudden cardiac death | m | 38 | 24 | <LOQ | 120.894 | 9.123 | 1.000 | 0.076 |
| 39 | Traffic accident (car driver, multiple injuries) | m | 32 | 30 | 0.59 | 89.893 | 7.120 | 1.000 | 0.080 |
| 40 | Work-related transport accident (pedestrian injured in collision with railway vehicle, multiple injuries) | m | 56 | 48 | <LOQ | 75.051 | 5.917 | 1.000 | 0.079 |
| 41 | Traffic accident (pedestrian injured in collision with car, multiple injuries) | m | 66 | 24 | <LOQ | 91.299 | 6.551 | 1.000 | 0.073 |
| 42 | Diabetic coma with ketoacidosis | m | 39 | 48 | <LOQ | 94.039 | 6.565 | 1.000 | 0.070 |
| 43 | Sudden cardiac death | m | 64 | 48 | <LOQ | 82.215 | 7.064 | 1.000 | 0.086 |
| 44 | Accidental fall from building | m | 21 | 64 | 1.07 | 97.001 | 7.170 | 1.000 | 0.078 |
| 45 | Assault by strangulation | f | 72 | 15 | 0.55 | 89.407 | 6.950 | 1.000 | 0.078 |
| 46 | Traffic accident (car driver, multiple injuries) | m | 57 | 48 | <LOQ | 88.098 | 6.251 | 1.000 | 0.073 |
| 47 | Traffic accident (car driver, multiple injuries) | m | 67 | 24 | <LOQ | 123.122 | 7.158 | 1.000 | 0.058 |
| 48 | Work-related injury of head | m | 59 | 24 | <LOQ | 84.961 | 6.333 | 1.000 | 0.076 |
| *Controls: ratio/median (q1, q3)* | | *21m/4f* | *57 (28, 61)* | *24 (15, 30)* | *0.00 (0.00, 0.00)* | *94.199 (89.407, 102.671)* | *7.734 (7.064, 8.368)* | *1.000 (1.000, 1.000)* | *0.079 (0.076, 0.088)* |
| Statistics | test | *χ^2^*-test | *U* | *U* | *U* | *U* | *U* | *U* | *U* |
|  | characteristic value | *χ^2^*= 0.610 | *Z* = 0.890 | *Z* = −0.790 | *Z* = 0.420 | *Z*= 0.230 | *Z* = 2.270 | *Z* = −0.230 | *Z* = 1.570 |
|  | *P* value | *0.435* | *0.373* | *0.429* | *0.675* | *0.818* | ***0.0232*** | *0.818* | *0.116* |
